# Beyond the viral triad: hantavirus cardiopulmonary syndrome as a blind spot in Brazil’s post-pandemic respiratory diagnostic reasoning

**DOI:** 10.1016/j.nmni.2026.101803

**Published:** 2026-06-23

**Authors:** Klinger Soares Faico-Filho, Nancy Bellei

**Affiliations:** a Universidade Federal de São Paulo (UNIFESP), Escola Paulista de Medicina (EPM), Departamento de Medicina, Disciplina de Clínica Médica e Medicina Laboratorial, Rua Pedro de Toledo, 781 – Vila Clementino, São Paulo, SP, CEP: 04024-002, Brazil; b Universidade Federal de São Paulo (UNIFESP), Escola Paulista de Medicina (EPM), Laboratório de Virologia, Departamento de Medicina, Divisão de Doenças Infecciosas, São Paulo, SP, Brazil

**Keywords:** Hantavirus, Hantavirus cardiopulmonary syndrome, Severe acute respiratory syndrome, Differential diagnosis, Epidemiological surveillance

Dear Editor,

In May 2026, the World Health Organization issued an alert regarding a multi-country cluster of hantavirus infection linked to a cruise ship travelling from Ushuaia, Argentina [1]. The episode drew global attention to hantavirus largely because of an exceptional feature: person-to-person transmission of Andes virus, the only hantavirus for which this route is documented. We wish to use this moment of visibility to draw attention to a very different and far less remarked problem: Brazil sustains the largest burden of hantavirus cardiopulmonary syndrome (HCPS) in the Americas through the conventional rodent-to-human route, and continues to lose patients not to an exotic mode of transmission but to a diagnosis that is made too late (see Table 1).

**Table 1:** The viral triad versus hantavirus in the diagnostic triage of severe acute respiratory syndrome in Brazil.

Dimension	Viral triad (influenza/RSV/SARS-CoV-2)	Hantavirus (HCPS)
Place in post-pandemic reasoning	Automatic differential	Frequently absent
Key trigger for suspicion	Seasonality; urban contact	Rural or rodent exposure — must be actively asked
Early laboratory clue	Respiratory viral panel	Thrombocytopenia with haemoconcentration
Capture in SIVEP-Gripe	Direct	Depends on active clinical suspicion
Case-fatality rate	Low to moderate	Approximately one third

The scale is substantial. Brazil reports the highest number of HCPS cases on the continent, with case-fatality rates around one third and a strong association with rural occupational exposure [2]. A recent epidemiological study from southern Brazil makes the core problem explicit: counterintuitively, patients who sought medical care early did not fare better, a finding the authors attribute to the initial difficulty of distinguishing hantavirus from other viral illnesses; conversely, regions with higher notification rates showed lower lethality, suggesting that surveillance capacity itself shapes outcome [2]. The bottleneck, in other words, is not how fast the patient reaches care, it is whether the clinician considers the diagnosis at all.

This is where a structural feature of Brazilian surveillance becomes relevant. Respiratory surveillance in Brazil is organized around influenza-like illness and severe acute respiratory infection (SARI), the latter under universal notification and reported through the SIVEP-Gripe platform [3]. Years of pandemic response have consolidated a respiratory diagnostic pathway built around a viral triad — influenza, respiratory syncytial virus, and SARS-CoV-2. HCPS presents as severe acute respiratory syndrome and would enter this very pathway; yet recognizing it depends on an epidemiological trigger (rural or rodent exposure) that must be actively sought, and on an early laboratory clue — thrombocytopenia with haemoconcentration — that is easily overlooked once a patient is on a standardized viral panel. We suggest, as a clinical hypothesis, that the post-pandemic narrowing of respiratory reasoning toward this triad has made hantavirus less likely to be considered, even where the robust SARI architecture would otherwise capture it.

Several concrete measures follow. First, in municipalities already mapped as vulnerable to HCPS — vulnerability indices exist in the Brazilian literature but are not integrated into respiratory surveillance [4] — the SARI investigation protocol could incorporate an explicit epidemiological trigger for rural and rodent exposure, prompting reflex hantavirus testing when SARI presents with thrombocytopenia and a negative triad panel. Second, these vulnerability maps should be linked to respiratory surveillance rather than maintained as a separate zoonosis silo. Third, continuing education should be directed specifically at regions of low notification, where low case counts reflect a diagnostic blind spot rather than true absence of disease.

The cruise-ship cluster reminded the world that hantavirus exists. Brazil, however, does not need an exceptional outbreak to act on the endemic and silent form of the disease that already kills roughly one patient in three. Integrating hantavirus into post-pandemic SARI reasoning — as a named entity in the diagnostic pathway, not an afterthought — is a low-cost intervention that could be in place before the next agricultural season, rather than the next ship, produces the cases that no one diagnoses in time.

## CRediT authorship contribution statement

**Klinger Soares Faico-Filho:** Conceptualization, Writing – original draft. **Nancy Bellei:** Writing – review & editing.

## Declaration of competing interest

The authors declare that they have no known competing financial interests or personal relationships that could have appeared to influence the work reported in this paper.
